# Asperienes A–D, Bioactive Sesquiterpenes from the Marine-Derived Fungus *Aspergillus flavus*

**DOI:** 10.3390/md17100550

**Published:** 2019-09-26

**Authors:** Yun-Feng Liu, Yu-Fei Yue, Li-Xi Feng, Hua-Jie Zhu, Fei Cao

**Affiliations:** 1 Key Laboratory of Medicinal Chemistry and Molecular Diagnostics of Education Ministry of China, Key Laboratory of Pharmaceutical Quality Control of Hebei Province, College of Pharmaceutical Sciences, Hebei University, Baoding 071002, China; liuyunfeng199011@163.com (Y.-F.L.); 13131148328@163.com (Y.-F.Y.); fenglixi888@163.com (L.-X.F.); 2 College of Life Sciences, Hebei University, Baoding 071002, China

**Keywords:** marine-derived fungus, *Aspergillus flavus*, sesquiterpene, bioactivity

## Abstract

Marine-derived fungi of the genera *Aspergillus* could produce novel compounds with significant bioactivities. Among these fungi, the strain *Aspergillus flavus* is notorious for its mutagenic mycotoxins production. However, some minor components with certain toxicities from *A. flavus* have not been specifically surveyed and might have potent biological activities. Our investigation of the marine-derived fungus *Aspergillus flavus* CF13-11 cultured in solid medium led to the isolation of four C-6′/C-7′ epimeric drimane sesquiterpene esters, asperienes A–D (**1**–**4**). Their absolute configurations were assigned by electronic circular dichroism (ECD) and Snatzke’s methods. This is the first time that two pairs of C-6′/C-7′ epimeric drimane sesquiterpene esters have successfully been separated. Aperienes A–D (**1**–**4**) displayed potent bioactivities towards four cell lines with the IC_50_ values ranging from 1.4 to 8.3 μM. Interestingly, compounds **1** and **4** exhibited lower toxicities than **2** and **3** toward normal GES-1 cells, indicating more potential for development as an antitumor agent in the future.

## 1. Introduction

Natural products from marine-derived fungi have been a topic of intensive research in drug discovery considering their pharmacological importance [1,2,3]. Among these bioactive compounds, sesquiterpenes (including drimane sesquiterpenes, phenolic bisabolane sesquiterpenes, eremophilane sesquiterpenes, cyclonerodiol sesquiterpenes, and so on) are produced by some marine-derived fungal species mainly from the genera *Aspergillus* [4,5,6], *Trichothecium* [7], *Graphostroma* [8], and *Trichoderma* [9]. These compounds display a diverse array of biological activities, including cytotoxic [6], antibacterial [4], nitric oxide inhibitory [5], nematicidal [7], anti-inflammatory [8], and growth inhibition of marine phytoplankton activities [9], which continues to stimulate efforts to discover new sesquiterpenes from nature. Drimane sesquiterpenes, as an important family of sesquiterpenes, showed potent cytotoxic activities against a wide cell variety, including HL-60, SMMC-721, A-549, ACHN, HCT-15, MDA-MB-231, NCI-H23, NUGC-3, and PC-3 cells [10,11].

**Figure 1:**
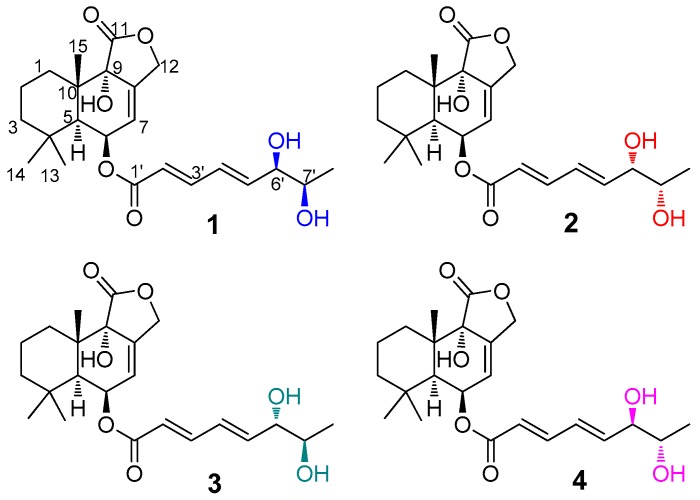
Chemical structures of **1**–**4** (color designations mean on the –OH)**.**

In our continuing efforts to identify new bioactive secondary metabolites from marine-derived fungi *Aspergillus* spp. [12,13,14], bioactive investigations on the extract of the fungal strain *Aspergillus flavus* CF13-11 showed significant cytotoxic activity. Marine-derived *Aspergillus flavus* is well known for its production of mutagenic mycotoxins and other bioactive compounds (meroterpenoids, indole-diterpenoids, and so on) [15,16]. Our chemical investigations of the fungus CF13-11 using a bioassay-guided method led to the isolation of four toxic C-6/C-7 epimeric drimane sesquiterpene esters, asperienes A–D (**1**–**4**) (Figure 1), whose structures were established on the basis of spectroscopic analysis and supported by electronic circular dichroism (ECD) and Snatzke’s analysis. Herein, we report the isolation, structural elucidation and bioactivities of these compounds.

## 2. Results

**Figure 2:**
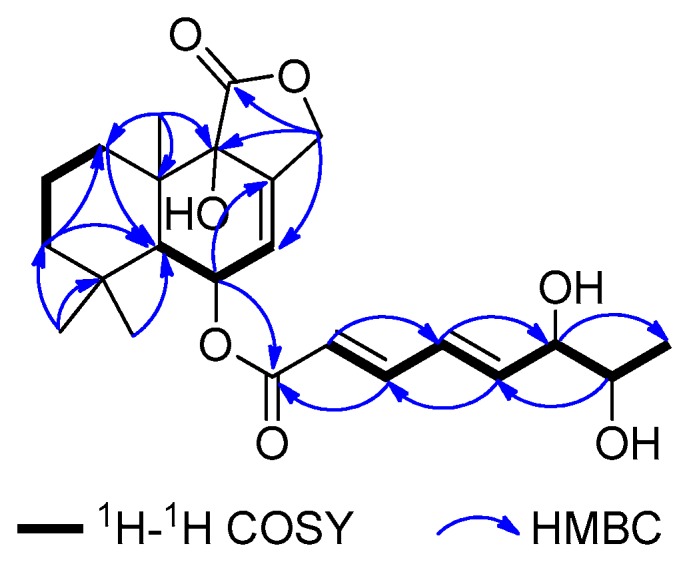
COSY and key HMBC correlations of **1**–**4**.

Asperiene A (**1**) (Figure 1) was isolated as a yellow oil with the molecular formula C_23_H_32_O_7_ (eight degrees of unsaturation) by its HR-ESI-MS spectrum. The signals in the 1D spectra of **1** (Table 1 and Table 2, Figures S1 and S2) indicated the presence of two ester carbonyl groups (*δ*_C_ 174.4 C, and 165.4 C), two disubstituted double bonds (*δ*_H_ 7.24 dd, 6.47 dt, 6.33 m, and 5.95 d; *δ*_C_ 145.3 CH, 145.3 CH, 127.3 CH, and 119.8 CH), and one trisubstituted double bond (*δ*_H_ 5.80 s, *δ*_C_ 136.6 C, and 121.4 CH) in **1**. These functional groups occupied five of eight degrees of unsaturation in **1**, suggesting a tricyclic framework for it. Moreover, three methyl signals with a singlet (*δ*_H_ 1.07 s, 1.07 s, and 0.93 s; 32.1 CH_3_, 24.3 CH_3_, and 18.3 CH_3_) were also observed in the ^1^H and ^13^C NMR spectra of **1**. The above characteristic NMR data revealed that **1** should be a drimane-type sesquiterpene with a lactone ring [17,18,19]. Comparison of the NMR data of **1** with those of strobilactone B from the marine-derived fungus *Aspergillus ustus* [20] suggested that **1** shares a similar nucleus structure with the known compound strobilactone B. The main difference between them was the presence of the additional side-chain signals for (2′*E*,4′*E*)-5′-carboxypenta-2′,4′-dienoyl moiety (*δ*_H_ 7.24 (1H, dd, *J* = 15.6, 3.6 Hz, H-3′), 6.47 (1H, dt, *J* = 15.6, 3.6 Hz, H-4′), 6.33 (1H, m, H-5′), 3.98 (1H, brs, H-6′), 3.57 (1H, t, *J* = 6.0 Hz, H-7′), and 0.96 (3H, d, *J* = 6.0 Hz, H-8′); *δ*_C_ 165.4, 145.3, 145.3, 127.3, 119.8, 74.4, 69.3, and 18.2; ^1^H–^1^H COSY cross-peaks of H-2′/H-3′/H-4′/H-5′/H-6′/H-7′/H-8′ (Figure 2)) in **1**. The HMBC correlations from H-6 proton (*δ*_H_ 5.59) to the C-1′ ester carbonyl demonstrated that the side chain moiety was attached at C-6. Thus, the planar structure for **1** was identified, which was confirmed by a detailed analysis of the HSQC, ^1^H–^1^H COSY, and HMBC spectra (Figure 2 and Figures S3–S5) of **1**.

All of the compounds asperienes A–D (**1**–**4**) were obtained with the same molecular formula C_23_H_32_O_7_, and the almost identical 1D and 2D NMR data (Table 1 and Table 2, Figures S1–S27), suggesting that all of **1**–**4** may be epimers. Upon carefully comparing the ^13^C NMR spectra of **1**–**4** (Table 2), the signals, attributable to C-6′, C-7′, and C-8′ in the side chains were found to be different, indicating that **1**–**4** were stereoisomers differing in the configurations of C-6′ and C-7′.

The relative configurations of the sesquiterpenoidal nucleus of **1**–**4** were determined by their NOESY experiments (Figures S6, S13, S20 and S27), all of which showed NOE correlations from H-15 to H-14, from H-5/H-6 to H-13, and from 9-OH to H-5. The relative configurations of C-6′ and C-7′ in the side chain of **1**–**4** were assigned by NMR data analysis. The NMR data of the side chain for **1** and **2** ((*δ*_C_ 18.2 (C-8′), *δ*_H_ 3.98 (H-6′), and *δ*_H_ 3.57 (H-7′) for **1**; (*δ*_C_ 18.2 (C-8′), *δ*_H_ 3.96 (H-6′), and *δ*_H_ 3.57 (H-7′) for **2**) were indicative of an *erythro* configuration (6′*R*,7′*R* or 6′*S*,7′*S*) at C-6′ and C-7′ for **1** and **2**, according to the literature [20]. Whereas, those of **3** and **4** [(*δ*_C_ 19.2 (C-8′), *δ*_H_ 3.87 (H-6′), and *δ*_H_ 3.49 (H-7′) for **3**; (*δ*_C_ 19.3 (C-8′), *δ*_H_ 3.84 (H-6′), and *δ*_H_ 3.48 (H-7′) for **4**] hinted at presence of a *threo* configuration (6′*S*,7′*R* or 6′*R*,7′*S*) at C-6′ and C-7′ for **3** and **4** [20].

**Figure 3:**
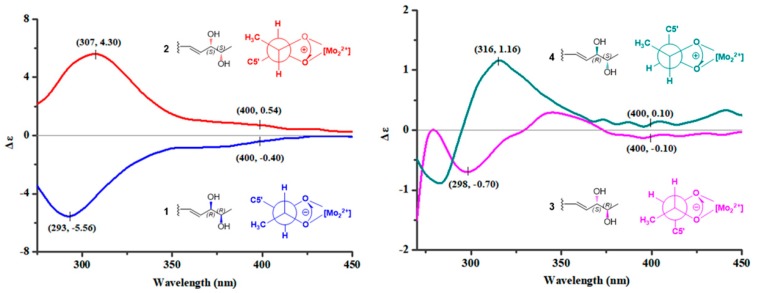
The electronic circular dichroism (ECD) spectra of Mo-complexes of **1**–**4**.

**Figure 4:**
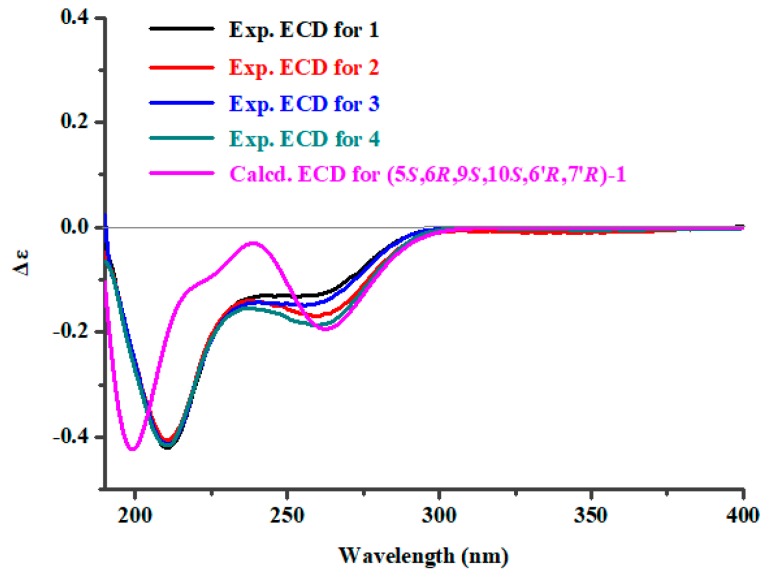
The calculated ECD spectrum of (5*S*,6*R*,9*S*,10*S*,6′*R*,7′*R*)-**1** and the experimental ECD spectra of **1**–**4**.

In order to assign the absolute configurations of C-6′ and C-7′ in **1**–**4**, induced circular dichroism (ICD) procedure (Snatzke’s method) was carried out for them [21,22]. The Mo-complexes of **1** (0.5 mg) and dimolybdenum tetraacetate (Mo_2_(OAc)_4_) were prepared and used to obtain its ICD spectrum. According to the ICD spectra of the reference Mo-complexes [21,22], the negative Cotton effect bands II (near 400 nm) and IV (around 293 nm) in the ICD spectrum of Mo-complexes of **1** (Figure 3) suggested the 6′*R*,7′*R* absolute configuration of **1**. Accordingly, the absolute configurations of 6′*S*,7′*S*, 6′*S*,7′*R*, and 6′*R*,7′*S* were assigned to compounds **2**, **3**, and **4**, respectively, by the same procedure (Figure 3). Then, electronic circular dichroism (ECD) method [23,24,25] was used to assign the absolute configuration of the sesquiterpenoidal nucleus for **1**–**4**. The possible structure (based on the relative configurations of the sesquiterpenoidal nucleus) (5*S*,6*R*,9*S*,10*S*,6′*R*,7′*R*)-**1** for **1** was used for ECD quantum chemical calculation and comparison of experimental ECD spectrum of **1**. ECD calculations for all of the stable conformers of (5*S*,6*R*,9*S*,10*S*,6′*R*,7′*R*)-**1** (Table S1) were performed at the B3LYP/6-311++G(2d,p)//B3LYP/6-311+G(d) level in the gas phase by time-dependent density functional theory (TDDFT). ECD simulation for (5*S*,6*R*,9*S*,10*S*,6′*R*,7′*R*)-**1** was calculated by Boltzmann statistics (σ 0.25 eV). It was shown that the calculated ECD spectrum of (5*S*,6*R*,9*S*,10*S*,6′*R*,7′*R*)-**1** matched well with the experimental ECD spectrum of **1** (Figure 4), indicating that the absolute configuration of **1** could be defined as 5*S*,6*R*,9*S*,10*S*,6′*R*,7′*R*. The absolute configurations of the sesquiterpenoidal nucleus in **2**–**4** could also be assigned as 5*S*,6*R*,9*S*,10*S*, due to the fact that the ECD spectra of **2**–**4** were in close and good agreement with that of **1** (Figure 4).

**Figure 5:**
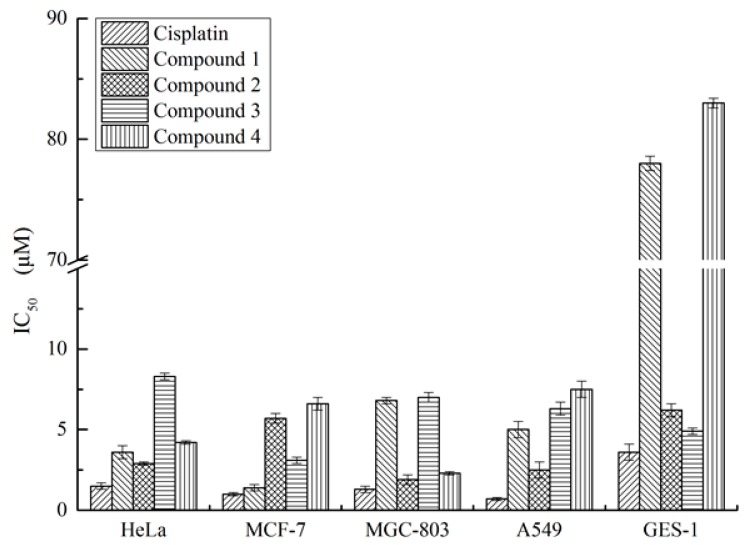
Cytotoxic activities of compounds **1**–**4**.

In previous literature, drimane sesquiterpene derivatives were reported to possess potent cytotoxic activities toward multiple tumor cell lines [26,27,28]. Therefore, the cytotoxic activities of **1**–**4** to four tumor cell lines (HeLa, MCF-7, MGC-803, and A549) were tested. All of the asperienes A–D (**1**–**4**) showed significant cytotoxic activities against these four cell lines (IC_50_, 1.4–8.3 μM) (Figure 5). Among them, compound **1** exhibited the strongest activity to MCF-7 cells with the IC_50_ value of 1.4 μM. Interestingly, the toxicities for **1**–**4** toward GES-1 cells were also examined, with the different IC_50_ values of 78, 6.2, 4.9, and 83 μM, respectively (Tables S2 and S3).

## 3. Discussion

Drimane sesquiterpene derivatives are a structurally variable family of natural terpenoids mainly obtained from fungi and plants [29]. However, many derivatives of these drimane sesquiterpenoids were esterified at C-6 with fatty acyl moieties, which possibly contained more than one stereogenic carbon [20,30]. These stereogenic carbons in the side chains of the drimane sesquiterpenoids resulted in the presence of many epimers, which were difficult to separate from each other due to their close polarity. Further, it was difficult to determine the absolute configurations of these stereogenic carbons due to the high conformational flexibility of the chains in them. In fact, two of four sesquiterpenoidal epimers (**1**–**4**) have been previously obtained from an algicolous *Aspergillus ustus* as inseparable epimeric mixtures, whose absolute configurations were also not assigned [30]. In the course of our study, the epimers **1**–**4** were firstly obtained as a mixture with a ratio of 1:1:1:1. In order to separate these two pair of epimers, HPLC separation was tried on Daicel Chiralpak IA, IB, IC, ID, OB, and OD columns with different mobile phases, including various combinations of n-hexane/IPA or n-hexane/EtOH solvents. Fortunately, **1**–**4** were separated successfully from each other by using n-hexane/EtOH (78:22) on Chiralpak IC column. Further, in the present research, Snatzke’s method was used as a powerful chemical approach for the configurational investigation of these four sesquiterpenoidal epimers (**1**–**4**).

*Aspergillus flavus* is well known as a saprotrophic fungus that infects and contaminates preharvest and postharvest seed crops, such as corn (maize), rice, and peanut (groundnuts) [31,32]. *A. flavus* infections are notorious for carcinogenic and mutagenic mycotoxin production, which causes enormous agricultural economic loss and poses significant risk to both humans and domestic animals [33,34]. Among these mycotoxins, the aflatoxins produced by *A.* *flavus* have been extensively studied and proven to be major risk factors for hepatocellular carcinoma or aspergillosis in Southeast Asia and Africa [35]. However, the fungus *A. flavus* also could generate the other structurally versatile mycotoxins because it consists of a large number of biosynthetic gene clusters [36]. These diversified mycotoxins from the fungus *A. flavus*, which are minor components but have certain toxicities, may not have been specifically surveyed and may have potent biological activities. Thus, the purpose of this research was to isolate and characterize new toxic natural products with potent bioactivities from the marine-derived fungal strain *A.* *flavus* CF13-11 to shed much light on a better understanding of the mycotoxins produced by *A.* *flavus*. The results suggested that the configuration at C-6′ in **1**–**4** may play an important role for the toxicity. The results also indicated that compounds **1** and **4** with higher activities and lower toxicities might have more potential for the development of an antitumor agent, but compounds **2** and **3** were the minor mycotoxins produced by *A.* *flavus*.

## 4. Experimental Section 

### 4.1. General Experimental Procedures

All of the experimental procedures were the same as in our previously reported work [12].

### 4.2. Isolation of the Fungal Material

The fungus CF13-11 was isolated from marine sediment collected from the Bohai Sea in July 2015. This strain, which was identified as *Aspergillus flavus* according to its 16*S* rRNA amplification and sequencing of the ITS region (Genbank, KY979507), was deposited in College of Pharmaceutical Sciences, Hebei University, China. The fermentation (thirty 1-L Erlenmeyer flasks) of the fungus *A. flavus* CF13-11 was carried out using solid culture (100 g rice and 100 mL water in each Erlenmeyer flask) at 28 °C for 25 days. The fermented medium was extracted with MeOH six times to obtain the crude extract (28.0 g). The crude extract was then partitioned between EtOAc and H_2_O to give the EtOAc extract (13.0 g), which was subjected to vacuum silica gel column chromatography with a CH_2_Cl_2_/MeOH (100:0, 80:1, 40:1, 20:1, 10:1, 5:1, and 1:1) gradient system to offer seven fractions (F.1–F.7). Among these fractions, F.3 (2.3 g) was separated by using silica gel column chromatography with a petroleum ether/EtOAc gradient system (2:1, 1:1, and 1:2) to offer three subfractions (F.3.1–F.3.3). Then, subfraction F.3.2 (95 mg) with certain toxicity was purified by ODS silica gel, Sephadex LH-20, and semi-preparative HPLC (Waters C_18_ column (5 μm, 10 × 250 mm), 2.0 mL/min, MeOH/H_2_O (70:30)) to give the mixture of compounds **1**–**4** (26.2 mg). Finally, the epimeric mixture **1**/**2**/**3**/**4** was successfully separated by semi-preparative HPLC on a Daicel Chiralpak IC column using n-hexane/EtOH (78:22) at a flow rate of 2.0 mL/min, affording compounds **1** (4.6 mg), **2** (4.4 mg), **3** (4.8 mg), and **4** (4.5 mg).

Asperiene A (**1**): yellow oil. $[\alpha {]}_{D}^{20}$ −180 (*c* 0.4, MeOH); UV (MeOH) *λ*_max_(log ԑ): 261 (2.13) nm; CD (MeOH), λ_max_ (Δ*ε*) 211 (‒0.42), 257 (‒0.13) nm; IR (KBr), *v*_max_ 3436, 2925, 1775, 1706, 1632, 1451, 1138, 759 cm^‒1^; ^1^H and ^13^C NMR data, see Table 1 and Table 2; HRESIMS m/z 443.2030 [M + Na]^+^ (calcd. for C_23_H_32_NaO_7_, 443.2040).

Asperiene B (**2**): yellow oil. $[\alpha {]}_{D}^{20}$ −203 (*c* 0.4, MeOH); UV (MeOH) *λ*_max_(log ԑ): 261 (2.10) nm; CD (MeOH), λ_max_ (Δ*ε*) 210 (‒0.41), 258 (‒0.17) nm; IR (KBr), *v*_max_ 3435, 2925, 1776, 1702, 1629, 1449, 1140, 762 cm^−1^; ^1^H and ^13^C NMR data, see Table 1 and Table 2; HRESIMS m/z 443.2035 [M + Na]^+^ (calcd. for C_23_H_32_NaO_7_, 443.2040).

Asperiene C (**3**): yellow oil. $[\alpha {]}_{D}^{20}$ −194 (*c* 0.4, MeOH); UV (MeOH) *λ*_max_(log ԑ): 261 (2.16) nm; CD (MeOH), λ_max_ (Δ*ε*) 211 (‒0.41), 259 (−0.15) nm; IR (KBr), *v*_max_ 3436, 2923, 1775, 1703, 1637, 1451, 1138, 759 cm^−1^; ^1^H and ^13^C NMR data, see Table 1 and Table 2; HRESIMS m/z 443.2027 [M + Na]^+^ (calcd. for C_23_H_32_NaO_7_, 443.2040).

Asperiene D (**4**): yellow oil. $[\alpha {]}_{D}^{20}$ ‒217 (*c* 0.4, MeOH); UV (MeOH) *λ*_max_(log ԑ): 261 (2.14) nm; CD (MeOH), λ_max_ (Δ*ε*) 210 (−0.42), 259 (−0.19) nm; IR (KBr), *v*_max_ 3434, 2923, 1775, 1706, 1632, 1452, 1141, 762 cm^−1^; ^1^H and ^13^C NMR data, see Table 1 and Table 2; HRESIMS m/z 443.2029 [M + Na]^+^ (calcd. for C_23_H_32_NaO_7_, 443.2040).

### 4.3. General Computational Procedure

The molecular model of (5*S*,6*R*,9*S*,10*S*,6′*R*,7′*R*)-**1** was built up and performed for conformational search using MMFF94S force field (BARISTA software, CONFLEX Corporation). Within a 10.0 kcal/mol relative energy window, 120 stable conformers for (5*S*,6*R*,9*S*,10*S*,6′*R*,7′*R*)-**1** were given, which were then performed for structural optimizations at the B3LYP/6-311 + G(d) levels by density functional theory method. ECD calculations for all of the optimized conformers were carried out at the B3LYP/6-311++G(2d,p) level in gas-phase by time-dependent density functional theory (TD-DFT) with a total of 60 excited states. All of the above calculations were performed with the Gaussian 09 package (Gaussian Inc., Wallingford, CT). Finally, a standard deviation of 0.25 eV was applied for ECD simulations for (5*S*,6*R*,9*S*,10*S*,6′*R*,7′*R*)-**1** to give its calculated ECD spectrum.

### 4.4. Snatzke’s Method

The ECD spectra of compounds **1**–**4** were firstly recorded to set up as a background spectrum, and then the ICD spectra of Mo-complexes of **1**–**4** were offered according to the published procedure [37]. 

### 4.5. Biological Assays

The isolated compounds (**1**–**4**) and the positive control cisplatin against a panel of cell lines were evaluated for their cytotoxicities in vitro by using MTT method [38], including human tumor cell lines, HeLa (cervical cancer), MCF-7 (breast cancer), MGC-803 (gastric cancer), and A549 (lung cancer), together with a non-tumoral cell line, GES-1 (human gastric epithelium). Cells were plated in 96-well plates at a density of 4000 cells (in 100 μL of culture medium) per well (well growth area 0.32 cm^2^). Each tumor cell line was exposed to each test compound at various concentrations in triplicate for 48 h, with cisplatin used as positive control substances.

## 5. Conclusions

Four toxic C-6/C-7 epimeric drimane sesquiterpene esters, asperienes A–D (**1**–**4**) (Figure 1), were obtained from the fungus *A. flavus* CF13-11. ECD and Snatzke’s methods were applied to assign the absolute configurations of **1**–**4**. Cell models, including four tumor cells and one normal cell, were used to evaluate the toxicities and bioactivities of **1**–**4**. This work suggested that the minor mycotoxins produced by *A. flavus* may have potent biological activities.

## Figures and Tables

**Table 1:** ^1^H NMR data (*δ*) of **1–4** (600 MHz, DMSO-*d*_6_, *δ* in ppm, *J* in Hz).

Position	1	2	3	4
1	1.98, dt (13.2, 3.6)	1.98, dt (13.2, 3.6)	1.98, dt (13.2, 4.2)	1.98, dt (13.2, 4.2)
	1.84, d (13.2)	1.84, d (13.2)	1.84, d (13.2)	1.82, d (13.2)
2	1.61, dd (13.8, 13.2)	1.61, dd (13.8, 13.2)	1.61, m	1.60, m
	1.48, d (13.2)	1.48, d (13.2)	1.48, d (13.2)	1.48, d (12.6)
3	1.35, d (13.2)	1.35, d (13.2)	1.35, d (13.2)	1.35, d (12.6)
	1.21, m	1.22, m	1.23, d (13.2)	1.21, d (12.6)
5	2.02, d (3.6)	2.02, d (3.6)	2.02, d (3.6)	2.02, d (3.6)
6	5.59, s	5.59, s	5.59, s	5.59, s
7	5.80, s	5.80, s	5.80, s	5.80, s
12	4.79, d (12.6)	4.79, d (13.2)	4.79, d (12.6)	4.80, d (12.6)
	4.89, d (12.6)	4.89, d (13.2)	4.89, d (12.6)	4.89, d (12.6)
13	0.93, s	0.93, s	0.93, s	0.93, s
14	1.07, s	1.07, s	1.07, s	1.07, s
15	1.07, s	1.07, s	1.07, s	1.07, s
2′	5.95, d (15.6)	5.95, d (13.2)	5.95, d (15.6)	5.95, d (12.6)
3′	7.24, dd (15.6, 3.6)	7.24, dd (13.2, 3.6)	7.23, dd (15.6, 3.6)	7.22, t (12.6)
4′	6.47, td (15.6, 3.6)	6.47, t (13.2)	6.44, td (15.6, 4.8)	6.43, t (12.6)
5′	6.33, m	6.30, dd (13.2, 3.6)	6.36, dd (15.6, 4.8)	6.33, m
6′	3.98, brs	3.96, t (6.0)	3.87, brs	3.84, brs
7′	3.57, t (6.0)	3.57, t (5.4)	3.49, brs	3.48, brs
8′	0.96, d (6.0)	0.96, d (6.0)	1.04, d (6.0)	1.03, d (6.0)
9-OH	6.30, brs	6.29, brs	6.31, brs	6.31, brs

**Table 2:** ^13^C NMR data (*δ*) of **1**–**4** (150 MHz, DMSO-*d*_6_, *δ* in ppm).

Position	1	2	3	4
1	29.6, CH_2_	29.6, CH_2_	29.6, CH_2_	29.6, CH_2_
2	17.4, CH_2_	17.5, CH_2_	17.4, CH_2_	17.4, CH_2_
3	44.4, CH_2_	44.4, CH_2_	44.4, CH_2_	44.4, CH_2_
4	33.3, C	33.3, C	33.3, C	33.3, C
5	44.2, CH	44.2, CH	44.2, CH	44.2, CH
6	65.8, CH	65.8, CH	65.8, CH	65.8, CH
7	121.4, CH	121.4, CH	121.4, CH	121.4, CH
8	136.6, C	136.6, C	136.6, C	136.6, C
9	73.1, C	73.1, C	73.1, C	73.1, C
10	37.3, C	37.3, C	37.3, C	37.3, C
11	174.4, C	174.4, C	174.4, C	174.4, C
12	68.2, CH_2_	68.2, CH_2_	68.2, CH_2_	68.2, CH_2_
13	32.1, CH_3_	32.1, CH_3_	32.1, CH_3_	32.1, CH_3_
14	24.3, CH_3_	24.3, CH_3_	24.3, CH_3_	24.3, CH_3_
15	18.3, CH_3_	18.2, CH_3_	18.3, CH_3_	18.3, CH_3_
1′	165.4, C	165.4, C	165.4, C	165.4, C
2′	119.8, CH	119.9, CH	119.9, CH	120.0, CH
3′	145.3, CH	145.2, CH	145.4, CH	145.4, CH
4′	127.3, CH	127.3, CH	126.9, CH	127.1, CH
5′	145.3, CH	145.3, CH	146.1, CH	146.0, CH
6′	74.4, CH	74.6, CH	74.9, CH	75.1, CH
7′	69.3, CH	69.3, CH	69.6, CH	69.6, CH
8′	18.2, CH_3_	18.2, CH_3_	19.2, CH_3_	19.3, CH_3_

## Supplementary Materials

The following are available online at https://www.mdpi.com/1660-3397/17/10/550/s1, Figure S1: ^1^H NMR (600 MHz, DMSO-*d*_6_) spectrum of compound **1**, Figure S2: ^13^C NMR (600 MHz, DMSO-*d*_6_) spectrum of compound **1**, Figure S3: HSQC (DMSO-*d*_6_) spectrum of compound **1**, Figure S4: ^1^H-^1^H COSY (DMSO-*d*_6_) spectrum of compound **1**, Figure S5: HMBC (DMSO-*d*_6_) spectrum of compound **1**, Figure S6: NOESY (DMSO-*d*_6_) spectrum of compound **1**, Figure S7: HRESIMS spectrum of compound **1**, Figure S8: ^1^H NMR (600 MHz, DMSO-*d*_6_) spectrum of compound **2**, Figure S9: ^13^C NMR (600 MHz, DMSO-*d*_6_) spectrum of compound **2**, Figure S10: HSQC (DMSO-*d*_6_) spectrum of compound **2**, Figure S11: ^1^H-^1^H COSY (DMSO-*d*_6_) spectrum of compound **2**, Figure S12: HMBC (DMSO-*d*_6_) spectrum of compound **2**, Figure S13: NOESY (DMSO-*d*_6_) spectrum of compound **2**, Figure S14: HRESIMS spectrum of compound **2**, Figure S15: ^1^H NMR (600 MHz, DMSO-*d*_6_) spectrum of compound **3**, Figure S16: ^13^C NMR (600 MHz, DMSO-*d*_6_) spectrum of compound **3**, Figure S17: HSQC (DMSO-*d*_6_) spectrum of compound **3**, Figure S18: ^1^H-^1^H COSY (DMSO-*d*_6_) spectrum of compound **3**, Figure S19: HMBC (DMSO-*d*_6_) spectrum of compound **3**, Figure S20: NOESY (DMSO-*d*_6_) spectrum of compound **3**, Figure S21: HRESIMS spectrum of compound **3**, Figure S22: ^1^H NMR (600 MHz, DMSO-*d*_6_) spectrum of compound **4**, Figure S23: ^13^C NMR (600 MHz, DMSO-*d*_6_) spectrum of compound **4**, Figure S24: HSQC (DMSO-*d*_6_) spectrum of compound **4**, Figure S25: ^1^H-^1^H COSY (DMSO-*d*_6_) spectrum of compound **4**, Figure S26: HMBC (DMSO-*d*_6_) spectrum of compound **4**, Figure S27: NOESY (DMSO-*d*_6_) spectrum of compound **4**, Figure S28: HRESIMS spectrum of compound **4**, Table S1: The coordinate for the lowest-energy conformer [(5*S*,6*R*,9*S*,10*S*,6′*R*,7′*R*)-1] in ECD calculation, Table S2: Cytotoxic activities of compounds **1**–**4**, Table S3: Detailed cytotoxic data of compounds **1**–**4**.
