# Supplementary material for: Asperienes A–D, Bioactive Sesquiterpenes from the Marine-Derived Fungus Aspergillus flavus
Source: Mar Drugs. 2019 Sep 26;17(10):550. doi: 10.3390/md17100550 (PMC6836145; doi:10.3390/md17100550)
Supplement: Supplementary file 1 [file marinedrugs-17-00550-s001.zip › SI20190907.doc]

**List of Supporting Information**

**Figure S1.** ^1^H NMR (600 MHz, DMSO-*d*_6_) spectrum of compound **1**

**Figure S2.** ^13^C NMR (600 MHz, DMSO-*d*_6_) spectrum of compound **1**

**Figure S3.** HSQC (DMSO-*d*_6_) spectrum of compound **1**

**Figure S4.** ^1^H-^1^H COSY (DMSO-*d*_6_) spectrum of compound **1**

**Figure S5.** HMBC (DMSO-*d*_6_) spectrum of compound **1**

**Figure S6.** NOESY (DMSO-*d*_6_) spectrum of compound **1**

**Figure S7.** HRESIMS spectrum of compound **1**

**Figure S8.** ^1^H NMR (600 MHz, DMSO-*d*_6_) spectrum of compound **2**

**Figure S9.** ^13^C NMR (600 MHz, DMSO-*d*_6_) spectrum of compound **2**

**Figure S10.** HSQC (DMSO-*d*_6_) spectrum of compound **2**

**Figure S11.** ^1^H-^1^H COSY (DMSO-*d*_6_) spectrum of compound **2**

**Figure S12.** HMBC (DMSO-*d*_6_) spectrum of compound **2**

**Figure S13.** NOESY (DMSO-*d*_6_) spectrum of compound **2**

**Figure S14.** HRESIMS spectrum of compound **2**

**Figure S15.** ^1^H NMR (600 MHz, DMSO-*d*_6_) spectrum of compound **3**

**Figure S16.** ^13^C NMR (600 MHz, DMSO-*d*_6_) spectrum of compound **3**

**Figure S17.** HSQC (DMSO-*d*_6_) spectrum of compound **3**

**Figure S18.** ^1^H-^1^H COSY (DMSO-*d*_6_) spectrum of compound **3**

**Figure S19.** HMBC (DMSO-*d*_6_) spectrum of compound **3**

**Figure S20.** NOESY (DMSO-*d*_6_) spectrum of compound **3**

**Figure S21.** HRESIMS spectrum of compound **3**

**Figure S22.** ^1^H NMR (600 MHz, DMSO-*d*_6_) spectrum of compound **4**

**Figure S23.** ^13^C NMR (600 MHz, DMSO-*d*_6_) spectrum of compound **4**

**Figure S24.** HSQC (DMSO-*d*_6_) spectrum of compound **4**

**Figure S25.** ^1^H-^1^H COSY (DMSO-*d*_6_) spectrum of compound **4**

**Figure S26.** HMBC (DMSO-*d*_6_) spectrum of compound **4**

**Figure S27.** NOESY (DMSO-*d*_6_) spectrum of compound **4**

**Figure S28.** HRESIMS spectrum of compound **4**

**Table S1.** The coordinate for the lowest-energy conformer [(5*S*,6*R*,9*S*,10*S*,6′*R*,7′*R*)-**1**] in ECD calculation

**Table S2.** Cytotoxic activities of compounds **1**–**4**

**Table S3.** Detailed cytotoxic data of compounds **1**–**4**

**Figure S1.** ^1^H NMR (600 MHz, DMSO-*d*_6_) spectrum of compound **1**

**Figure S2.** ^13^C NMR (600 MHz, DMSO-*d*_6_) spectrum of compound **1**

**Figure S3.** HSQC (DMSO-*d*_6_) spectrum of compound **1**

**Figure S4.** ^1^H-^1^H COSY (DMSO-*d*_6_) spectrum of compound **1**

**Figure S5.** HMBC (DMSO-*d*_6_) spectrum of compound **1**

**Figure S6.** NOESY (DMSO-*d*_6_) spectrum of compound **1**

**Figure S7.** HRESIMS spectrum of compound **1**

**Figure S8.** ^1^H NMR (600 MHz, DMSO-*d*_6_) spectrum of compound **2**

**Figure S9.** ^13^C NMR (600 MHz, DMSO-*d*_6_) spectrum of compound **2**

**Figure S10.** HSQC (DMSO-*d*_6_) spectrum of compound **2**

**Figure S11.** ^1^H-^1^H COSY (DMSO-*d*_6_) spectrum of compound **2**

**Figure S12.** HMBC (DMSO-*d*_6_) spectrum of compound **2**

**Figure S13.** NOESY (DMSO-*d*_6_) spectrum of compound **2**

**Figure S14.** HRESIMS spectrum of compound **2**

**Figure S15.** ^1^H NMR (600 MHz, DMSO-*d*_6_) spectrum of compound **3**

**Figure S16.** ^13^C NMR (600 MHz, DMSO-*d*_6_) spectrum of compound **3**

**Figure S17.** HSQC (DMSO-*d*_6_) spectrum of compound **3**

**Figure S18.** ^1^H-^1^H COSY (DMSO-*d*_6_) spectrum of compound **3**

**Figure S19.** HMBC (DMSO-*d*_6_) spectrum of compound **3**

**Figure S20.** NOESY (DMSO-*d*_6_) spectrum of compound **3**

**Figure S21.** HRESIMS spectrum of compound **3**

**Figure S22.** ^1^H NMR (600 MHz, DMSO-*d*_6_) spectrum of compound **4**

**Figure S23.** ^13^C NMR (600 MHz, DMSO-*d*_6_) spectrum of compound **4**

**Figure S24.** HSQC (DMSO-*d*_6_) spectrum of compound **4**

**Figure S25.** ^1^H-^1^H COSY (DMSO-*d*_6_) spectrum of compound **4**

**Figure S26.** HMBC (DMSO-*d*_6_) spectrum of compound **4**

**Figure S27.** NOESY (DMSO-*d*_6_) spectrum of compound **4**

**Figure S28.** HRESIMS spectrum of compound **4**

**Table S1.** The coordinate for the lowest-energy conformer [(5*S*,6*R*,9*S*,10*S*,6′*R*,7′*R*)-**1**] in ECD calculation

----------------------------------------------------------------------------------

Coordinates (Angstroms)

X	Y	Z

-----------------------------------------------------------------------------------

C	3.87881600	2.74268300	1.39104700

C	3.22816300	3.30318200	0.12751600

C	2.02875800	2.45888300	-0.37021000

C	2.53761400	0.97919500	-0.56605500

C	3.37202600	0.33697200	0.60787500

C	4.43292100	1.34236200	1.12391000

C	1.50979000	0.00964400	-1.19032100

C	1.99581700	-1.41043800	-1.29601400

C	3.14919700	-1.80244700	-0.77594200

C	4.09139100	-0.89260600	-0.04282200

C	3.71142700	-3.19120100	-0.67284000

O	4.54496600	-3.15893800	0.51427300

O	0.27034500	-0.03537300	-0.41066100

C	2.51887500	-0.15948000	1.79477200

H	3.27892300	1.07840500	-1.36990100

O	5.10606900	-0.52336800	-1.00498200

C	0.84857900	2.66854200	0.60526600

C	1.59733700	3.02162300	-1.74053000

C	-2.02554500	-0.32392700	-0.15953000

C	-3.27424300	-0.54693000	-0.60836700

C	-4.45321000	-0.57326700	0.22944500

C	-5.68575900	-0.78926400	-0.25568500

C	-6.93341700	-0.83061600	0.57154600

C	-7.91295200	0.32808200	0.25161800

C	-8.45942600	0.32067900	-1.16807300

O	-6.60249400	-0.79553600	1.94721400

O	-8.97233600	0.29366500	1.22442400

C	-0.87686500	-0.31170400	-1.08233700

O	-0.92226000	-0.51323200	-2.27818100

C	4.79872100	-1.88799200	0.91041700

O	5.54012100	-1.63280700	1.81896500

H	3.16622700	2.73325000	2.22180800

H	4.69886600	3.39511800	1.71008300

H	3.98608800	3.35288500	-0.66595800

H	2.89055300	4.33267400	0.29152400

H	4.91223000	0.93271600	2.01620300

H	5.22141100	1.46190800	0.37291400

H	1.23596200	0.36436200	-2.18441800

H	1.32180100	-2.11217700	-1.78043400

H	2.95728600	-3.96520400	-0.52967200

H	4.35309300	-3.45854400	-1.51685800

H	2.03178100	0.66896900	2.30667000

H	3.14922200	-0.66137300	2.53264900

H	1.73442100	-0.84937000	1.48596100

H	5.88812500	-0.21185200	-0.52927200

H	-0.07086700	2.21346500	0.24439100

H	0.66361700	3.74260400	0.71195000

H	1.03415100	2.27447700	1.60347000

H	1.38513600	4.09208000	-1.65809100

H	2.38300000	2.90059700	-2.49323200

H	0.69016600	2.54543600	-2.12157300

H	-1.82057500	-0.14585000	0.89078600

H	-3.40498500	-0.71847200	-1.67519800

H	-4.33074800	-0.41895100	1.29724300

H	-5.81800600	-0.96722200	-1.32089400

H	-7.46240700	-1.77511100	0.34201700

H	-7.39306600	1.26747500	0.45661100

H	-9.18842800	1.12429000	-1.29267900

H	-7.66751100	0.47326400	-1.90442900

H	-8.95427000	-0.62942800	-1.40210700

H	-7.40461000	-0.53302400	2.42194800

H	-9.64995800	-0.33452300	0.94194000

-----------------------------------------------------------------------------------

Table S2. Cytotoxic activities of compounds 1–4

| **Compounds** | Cell lines IC_50_ (*μ*M) | | | |  | |
| --- | --- | --- | --- | --- | --- | --- |
|  | **HeLa** | **MCF-7** | **MGC-803** | **A549** | | **GES-1** |
| **1** | 3.6 ± 0.4 | 1.4 ± 0.2 | 6.8 ± 0.2 | 5.0 ± 0.5 | | 78 ± 5.9 |
| **2** | 2.9 ± 0.1 | 5.7 ± 0.3 | 1.9 ± 0.3 | 2.5 ± 0.5 | | 6.2 ± 0.4 |
| **3** | 8.3 ± 0.2 | 3.1 ± 0.2 | 7.0 ± 0.3 | 6.3 ± 0.4 | | 4.9 ± 0.2 |
| **4** | 4.2 ± 0.1 | 6.6 ± 0.4 | 2.3 ± 0.1 | 7.5 ± 0.5 | | 83 ± 0.5 |
| Cisplatin | 1.5 ± 0.2 | 1.0 ± 0.1 | 1.3 ± 0.2 | 0.7 ± 0.1 | | 3.6 ± 0.4 |

Table S3. Detailed cytotoxic data of compounds 1–4

| Compound |  | **HeLa** | **MCF-7** | **MGC-803** | **A549** | **GES-1** |
| --- | --- | --- | --- | --- | --- | --- |
| **1** |  | 3.23 | 1.28 | 6.57 | 5.16 | 78.27 |
|  |  | 3.56 | 1.45 | 6.88 | 5.43 | 72.43 |
|  |  | 4.03 | 1.59 | 6.88 | 4.52 | 84.23 |
|  | Standard deviation | 0.40 | 0.16 | 0.18 | 0.47 | 5.90 |
|  | Average | 3.61 | 1.44 | 6.78 | 5.04 | 78.31 |
| **2** |  | 3.03 | 5.39 | 1.83 | 2.89 | 6.29 |
|  |  | 2.90 | 5.56 | 1.66 | 2.70 | 6.62 |
|  |  | 2.79 | 6.03 | 2.23 | 2.03 | 5.91 |
|  | Standard deviation | 0.12 | 0.33 | 0.29 | 0.45 | 0.36 |
|  | Average | 2.91 | 5.66 | 1.91 | 2.54 | 6.27 |
| **3** |  | 8.23 | 3.15 | 7.13 | 6.30 | 5.13 |
|  |  | 8.56 | 3.26 | 7.34 | 6.69 | 4.66 |
|  |  | 8.13 | 2.95 | 6.79 | 5.97 | 5.03 |
|  | Standard deviation | 0.23 | 0.16 | 0.28 | 0.36 | 0.25 |
|  | Average | 8.31 | 3.12 | 7.09 | 6.32 | 4.94 |
| **4** |  | 4.26 | 6.86 | 2.23 | 7.20 | 83.13 |
|  |  | 4.35 | 6.23 | 2.46 | 7.30 | 83.30 |
|  |  | 4.13 | 6.83 | 2.18 | 8.13 | 84.03 |
|  | Standard deviation | 0.11 | 0.36 | 0.15 | 0.51 | 0.48 |
|  | Average | 4.25 | 6.64 | 2.29 | 7.54 | 83.49 |
| **Cisplatin** |  | 1.46 | 0.98 | 1.23 | 0.85 | 3.33 |
|  |  | 1.30 | 0.88 | 1.46 | 0.66 | 3.46 |
|  |  | 1.60 | 1.13 | 1.13 | 0.71 | 4.03 |
|  | Standard deviation | 0.15 | 0.13 | 0.17 | 0.10 | 0.37 |
|  | Average | 1.45 | 1.00 | 1.27 | 0.74 | 3.61 |
